# Validity and Reliability of the Korean Version of the Rushton Moral Resilience Scale–16™ in Nurses

**DOI:** 10.3390/healthcare14040424

**Published:** 2026-02-08

**Authors:** Eun A. Kim, Hae Ran Kim

**Affiliations:** 1 Department of Nursing, Honam University, Gwangju 58128, Republic of Korea; 2 Department of Nursing, College of Medicine, Chosun University, Gwangju 61452, Republic of Korea

**Keywords:** morality, resilience, psychological, burnout, professional, validation study, nurses

## Abstract

**Background/Objectives**: We aimed to evaluate the validity and reliability of the Korean version of the Rushton Moral Resilience Scale–16™ (RMRS-16™) for nurses. **Methods**: The RMRS-16™ was translated into Korean using a forward–backward translation process. Data were collected from 417 nurses working in five tertiary and three general hospitals in South Korea. Content validity was assessed using the content validity index. Construct validity was examined through exploratory and confirmatory factor analyses. Convergent validity was evaluated using Pearson correlation coefficients with resilience and known-groups validity was examined across burnout levels using analysis of variance and post hoc tests. Internal consistency reliability was assessed using Cronbach’s α. **Results**: Exploratory factor analysis identified four factors that explained 56.45% of the cumulative variance: response to moral adversity, relational integrity, personal integrity, and moral efficacy. Confirmatory factor analysis demonstrated an acceptable model fit (χ^2^/df = 1.77, standardized root mean square residual = 0.05, root mean square error of approximation = 0.06, goodness-of-fit index = 0.91, comparative fit index = 0.94, Tucker–Lewis index = 0.93), and both convergent and discriminant validity were supported. Moral resilience was positively correlated with resilience and differed significantly by burnout level. The total scale showed satisfactory internal consistency in the full sample (n = 417, Cronbach’s α = 0.86). **Conclusions**: The RMRS-16™ is a valid and reliable instrument for South Korean hospital practice. It can be used in intervention studies to assess and strengthen moral resilience in nursing practice.

## 1. Introduction

Nurses frequently encounter clinical situations that conflict with their moral values, including the provision of non-beneficial treatments, allocation of limited healthcare resources, and interpersonal conflicts within healthcare organizations [1,2]. In such contexts, nurses must navigate decisions that challenge their personal moral values due to institutional and environmental constraints. Consequently, nurses commonly experience moral conflict [3]. In morally challenging situations, moral injury may occur when nurses act in ways that contradict their moral standards, remain silent, comply with others’ decisions, or witness actions that violate their moral beliefs [4]. Exposure to morally injurious events that conflict with an individual’s belief system has been shown to result in psychological distress and discomfort [5]. For instance, during the COVID-19 pandemic, nurses reported experiencing moral injury after being unable to provide dignified care to critically ill patients or adequately support patients’ families, often accompanied by self-critical cognitions such as “I am not a good nurse” [4]. Similarly, nurses who witness futile treatments or diagnostic procedures may experience a loss of trust, wounded conscience, and perceived erosion of their core values [2].

Within hospital nursing organizations, conflicts among nurses are associated with experiences of indifference, betrayal, loss, hopelessness, and depressive symptoms, adversely affecting both individual nurses and care quality [6,7]. A systematic review reported that moral injury is consistently associated with adverse mental health outcomes, including helplessness and depression, across occupations and age groups, and contributes to negative consequences such as increased burnout and turnover intention [8,9]. In such circumstances, moral distress arises when nurses recognize the morally appropriate course of action but are constrained from acting upon it by institutional, procedural, or social factors. Moral distress threatens nurses’ core professional values and moral integrity and results in a range of negative outcomes [10].

Not all nurses, however, experience these adverse consequences to the same extent. Scholarly interest has increasingly focused on moral resilience as a construct that helps mitigate the negative effects of moral injury and distress by enabling individuals to navigate moral challenges and sustain moral functioning [11]. Moral resilience refers to the capacity to maintain or restore one’s moral values and integrity when confronted with moral adversity [12]. In this context, moral integrity is defined as the consistent enactment of what one believes to be morally right and good through one’s words and actions [13]. Importantly, moral resilience is considered an adaptive capacity, similar to general resilience, that can be strengthened through practice and training, thereby supporting recovery from moral injury and the sustained preservation of moral integrity [11,13]. Research on moral resilience has been conducted primarily in nursing and healthcare settings outside South Korea, whereas domestic studies remain limited and have largely focused on applications within moral education in the field of education [13,14].

In response to growing concerns about moral distress in clinical practice, the American Nurses Association (ANA) convened an expert panel in 2017 and has actively promoted policies and strategies aimed at strengthening moral resilience among clinical nurses [15]. The ANA reframed moral distress not as a condition to be eliminated but as an essential signal reflecting the moral values and commitments inherent in professional nursing practice, thereby shifting the focus toward enhancing moral resilience [15]. Furthermore, the ANA emphasized that strengthening moral resilience is necessary to reduce adverse outcomes associated with moral distress, including burnout, turnover, medical errors, and diminished quality of care, and underscored the importance of both individual and organizational engagement in this effort [15]. Since the COVID-19 pandemic, research examining nurses’ experiences of moral injury, distress, courage, and resilience has increased substantially [16,17]. Recent studies suggest that moral resilience functions as a protective factor, attenuating the negative effects of moral injury-related moral distress [17,18], mental health problems such as depression and anxiety [9,18], as well as burnout and turnover intention [19,20]. In South Korea, however, research involving nurses has primarily focused on moral distress [21] or moral courage [22], with limited attention to moral resilience. Accordingly, assessing nurses’ levels of moral resilience and developing targeted educational interventions may help mitigate the adverse effects of moral injury and moral distress.

The Rushton Moral Resilience Scale (RMRS), developed by Heinze et al. [9] for healthcare professionals in the United States, is a widely used instrument for measuring moral resilience. The RMRS has been translated and validated in several countries, including Saudi Arabia [23], China [24], Turkey [25], and Iran [26]. Although some versions retain the original factor structure and number of items, others have reported alternative factor structures or item reductions, suggesting cross-cultural variability. The original RMRS consists of 17 items across 4 factors—response to moral adversity, personal integrity, relational integrity, and moral efficacy—but has been criticized for the relatively low reliability of the personal integrity subscale (0.50) [9]. To address this limitation, Rushton et al. [27] refined the original scale and re-established its psychometric properties, resulting in the RMRS-16™ (Rushton Moral Resilience Scale–16™). The RMRS-16™ has subsequently been translated and validated in Greece [28] and Japan [29], with both versions retaining the four-factor, 16-item structure and demonstrating satisfactory reliability and validity. Given its robust psychometric properties and demonstrated cultural applicability, the RMRS-16™ was selected for adaptation in South Korea. Although moral resilience instruments have been validated in various countries, no study to date has examined the applicability of the RMRS-16™ in South Korean nursing practice.

In the South Korean nursing context, organizational cultures are often characterized by hierarchical structures and a strong emphasis on relational harmony, which may influence how moral resilience is experienced and expressed [6,7]. Within such environments, nurses may face additional challenges in expressing, negotiating, and sustaining their moral judgments during ethically challenging situations, particularly when these judgments conflict with organizational norms or authority [1,2,6]. Although moral distress and related ethical challenges among Korean nurses have been examined in prior studies [10,21,22], research focusing on nurses’ capacity to recover, adapt, and sustain moral integrity in response to moral adversity—that is, moral resilience—remains limited [13,14,21].

Within the broader literature on psychosocial well-being among healthcare professionals, moral resilience has been discussed alongside related constructs such as resilience, moral reasoning, ethical decision-making, and prosocial behavior, all of which have been identified as protective factors against work-related stress and burnout [11,16,20].

The purpose of this study was to adapt the RMRS-16™ [27] for use among South Korean nurses and to evaluate the validity and reliability of the Korean version of the scale (K-RMRS-16™).

## 2. Methods

### 2.1. Design and Participants

We employed a methodological design to translate the RMRS-16™ [9,27] into Korean and to evaluate the validity and reliability of the K-RMRS-16™.

To examine whether the factor structure derived from one sample could be replicated in an independent sample, it is recommended that exploratory factor analysis (EFA) and confirmatory factor analysis (CFA) be conducted using separate samples [30]. Additionally, methodological guidelines suggest a minimum sample size of 200 participants for each analysis or 10 participants per item for factor analysis [31]. Based on these recommendations and allowing for potential attrition, a total of 420 participants were recruited.

Participants were nurses who had worked for at least six months in tertiary or general hospitals and were recruited using convenience sampling. To minimize regional bias, hospitals were selected from four geographic regions in South Korea: the metropolitan area (Seoul and Gyeonggi), the central region (Daejeon and Chungnam), the Gyeongsang region (Busan and Daegu), and the Honam region (Gwangju and Jeonnam). Two hospitals from each region were included. Ultimately, data were collected from 420 nurses working in 5 tertiary and 3 general hospitals. After excluding incomplete or insincere responses, data from 417 participants were included in the final analysis (response rate: 99.3%; 40–65 participants per hospital).

Using random sampling procedures in SPSS, the total sample was divided into 2 independent subsamples: 208 and 209 participants for EFA and CFA, respectively.

### 2.2. Research Instruments

#### 2.2.1. Moral Resilience

Moral resilience was measured using the RMRS-16™, a revision of the 17-item RMRS [9,27]. In the revised version, 13 items from the original scale were retained, 4 were removed, and 3 were added. The RMRS-16™ consists of 16 items across 4 factors and measures respondents’ responses to challenging ethical situations encountered in their clinical practice over the past three months.

The first subscale, response to moral adversity, assesses emotional and behavioral responses when individuals are confronted with ethical challenges and includes experiences such as helplessness, feeling overwhelmed, persistent distress, and excessive self-sacrifice. This subscale consists of four reverse-scored items. The second subscale, relational integrity, refers to the ability or state of maintaining one’s moral beliefs while balancing personal values and relationships with others in situations of conflict. It includes maintaining one’s ethical stance despite relational pressures from supervisors or others and consists of four reverse-scored items. The third subscale, personal integrity, assesses the ability or state of acting consistently with one’s values and beliefs and maintaining ethical standards in one’s professional role, despite ethical challenges or adversity (four items). The fourth subscale, moral efficacy, refers to confidence in recognizing ethical issues and conflicts, communicating effectively about them, and resolving ethical challenges in ways that align with one’s values (four items) [9,27].

All items are rated on a 4-point Likert scale ranging from 1 (“strongly disagree”) to 4 (“strongly agree”). Eight items are reverse-scored, and higher total scores indicate higher levels of moral resilience. The original RMRS was validated among 702 healthcare professionals, including nurses and physicians, in the United States [9], whereas the RMRS-16™ was validated among 1297 healthcare professionals in Canada [27]. During scale development, the Cronbach’s α of the original RMRS was 0.84, with subscale reliabilities ranging from 0.50 to 0.78 [9]. For the RMRS-16™, overall reliability ranged from 0.85 to 0.86, with subscale reliabilities of 0.73–0.74 for response to moral adversity, 0.76–0.78 for personal integrity, 0.78 for relational integrity, and 0.72–0.76 for moral efficacy [27].

#### 2.2.2. Resilience

General resilience was measured using the Resilience Scale for Nurses developed by Park and Park [32], used with the authors’ permission. This instrument consists of 30 items across 5 factors: situational pattern (10 items), philosophical pattern (6 items), dispositional pattern (5 items), professional pattern (5 items) and relational pattern (4 items). Each item is rated on a 5-point Likert scale ranging from 1 (“not at all true”) to 5 (“very true”), with higher scores indicating greater resilience. At the time of development, the scale demonstrated excellent internal consistency (Cronbach’s α = 0.95) [32], and the Cronbach’s α in the present study was also 0.95.

#### 2.2.3. Burnout

Burnout was assessed using the burnout subscale of the Professional Quality of Life Scale, version 5, developed by Stamm [33]. The publicly available Korean version consisting of 10 items was used. Items are rated on a 5-point Likert scale ranging from 1 (“not at all”) to 5 (“very often”), with 5 items reverse-scored. Higher scores indicate higher levels of burnout. Based on T-score conversions provided by the instrument developer, scores of 43 or lower indicate low burnout, scores of 44–56 indicate moderate burnout, and scores of 57 or higher indicate high burnout [33]. The Cronbach’s α of the burnout subscale was 0.75 at the time of development [33] and 0.81 in the present study.

### 2.3. Procedure

#### 2.3.1. Instrument Permission and Translation

Permission to use and translate the RMRS-16™ was obtained from the Johns Hopkins School of Nursing by submitting a formal application through its official website. The translation and cultural adaptation process was conducted in accordance with World Health Organization guidelines [34].

First, two bilingual experts with doctoral degrees in nursing and psychology, both fluent in Korean and English and experienced in instrument development, independently performed forward translations from English to Korean. During the review and reconciliation stage, an expert panel consisting of the two translators and two members of the research team (four experts in total) compared the translated versions, reviewed item appropriateness, and reached consensus on wording and phrasing through discussion.

Subsequently, backward translation was independently conducted by a bilingual expert who is a professor of English literature at the authors’ affiliated university and who was blinded to the original English version. The expert panel compared the backward-translated version with the original instrument to evaluate semantic equivalence, conceptual clarity, cultural appropriateness, and discrepancies attributable to cultural differences, and revisions were made accordingly.

Particular attention was paid to the cultural context, considering the transition of South Korean nursing organizations from predominantly hierarchical structures toward more relationship-oriented cultures [35], to ensure measurement equivalence in the Korean version. For instance, in Item 14, the phrase “who have more authority than” was initially translated as “superiors” but was revised to “individuals who have greater authority than me” to better reflect relational dynamics. In Item 4, “conviction” was initially translated as “certainty” but was revised to “belief” to better align with value-related meaning. In Item 12, “I am confident in my ability to reason through ethical challenges” was initially translated literally but was revised to “I am confident in my ability to make rational judgments and resolve ethical challenges” to improve clarity and comprehension. Through these steps, a preliminary K-RMRS-16™ was finalized.

#### 2.3.2. Content Validity of the Preliminary Instrument

Content validity of the preliminary K-RMRS-16™ was evaluated by an expert panel consisting of six individuals: three nursing professors from universities in G city and three nurses with more than ten years of clinical experience and master’s degrees who were working at a tertiary hospital in G city. The experts were provided with the conceptual definition of moral resilience and asked to rate the relevance of each of the 16 items using a 4-point scale (1 = “not relevant,” 2 = “somewhat not relevant,” 3 = “relevant,” 4 = “highly relevant”). Qualitative feedback regarding item wording and clarity was also collected.

Content validity was assessed using the item-level content validity index, defined as the proportion of experts rating an item as 3 or 4, and the scale-level content validity index using the averaging method [36]. The item-level content validity index values ranged from 0.80 to 1.00 (criterion ≥ 0.78), and the scale-level content validity index using the averaging method was 0.97 (criterion ≥ 0.90), indicating satisfactory content validity. Based on expert feedback, terminology was refined for consistency (e.g., replacing “nurse” with “professional nurse” and “encounter” with “confront”). No items were deleted; wording revisions were made to 3 items, resulting in a 16-item preliminary instrument with 4 factors.

#### 2.3.3. Pilot Testing and Cognitive Interviewing

Pilot testing was conducted with 20 nurses, which falls within the recommended sample size of 20–40 participants for preliminary testing [37]. Participants were nurses who had worked for at least three months at a tertiary hospital in G city and who provided informed consent. Data were collected between 19 and 22 November 2024. Participants’ mean age was 36.7 years, and the average time required to complete the questionnaire was 2.71 min.

Cognitive interviews were conducted to assess cultural appropriateness, semantic equivalence, item comprehension, response difficulty, and clarity of content. No items were identified as requiring further revision. Internal consistency reliability of the preliminary instrument was satisfactory, with a Cronbach’s α of 0.89 for the total scale and subscale reliabilities of 0.88 for response to moral adversity, 0.75 for personal integrity, 0.87 for relational integrity, and 0.74 for moral efficacy. Based on these findings, the 16-item instrument was deemed appropriate for the main survey and was used without further modification.

#### 2.3.4. Psychometric Evaluation and Finalization of the K-RMRS-16™

Psychometric evaluation of the K-RMRS-16™ was conducted to examine its measurement properties. Structural validity, including factor structure, was assessed using EFA and CFA. Convergent validity and known-groups validity were examined by analyzing relationships between the K-RMRS-16™ and theoretically related constructs. Internal consistency reliability was evaluated to confirm the stability of the instrument. Based on these analyses, the final version of the K-RMRS-16™ was established.

### 2.4. Data Collection

Data collection was conducted sequentially between 19 November 2024 and 17 January 2025 across 4 geographic regions, with intervals of 10–12 days to ensure adequate regional representation.

Prior to data collection, institutional approval was obtained in accordance with each participating hospital’s administrative procedures. Recruitment announcements and survey links were posted through online communication channels of cooperating departments, allowing nurses to participate voluntarily. Written informed consent was obtained from all participants prior to participation. The online survey was designed to allow participants to proceed only after reviewing the study information sheet, confirming eligibility, and providing informed consent. If participants did not meet the inclusion criteria or did not agree to participate, the survey was automatically terminated.

All survey items were set as mandatory to allow final submission, and re-access to the survey after completion was restricted. The average time required to complete the questionnaire was approximately 15 min.

### 2.5. Ethical Considerations

This study was conducted in accordance with ethical standards and was approved by the Institutional Review Board of Honam University on 27 June 2024 (IRB No. 1041223-202406-HR-18). The study information sheet provided to participants included details regarding the purpose and procedures of the study, eligibility criteria, anonymity and confidentiality, potential benefits and risks of participation, voluntary participation, the right to withdraw at any time without penalty, and assurances that the collected data would be used solely for research purposes.

Participants were informed that all data would be stored in encrypted, password-protected files and securely retained, with all data to be destroyed within three years of study completion. Participants who completed the survey received a mobile gift voucher as compensation for their time. Any contact information collected for compensation purposes was deleted immediately after the voucher was issued.

### 2.6. Data Analysis

Data were analyzed using IBM SPSS Statistics version 29.0 and IBM AMOS version 29.0 (IBM Corp., Armonk, NY, USA). First, participants’ general characteristics and scale scores were analyzed using descriptive statistics. Homogeneity between the samples used for EFA and CFA was examined using independent-samples *t*-tests and chi-square tests.

Second, the instrument’s construct validity was evaluated through item analysis, factor analysis, convergent validity, and discriminant validity. Prior to conducting EFA (n = 208), item analysis was performed to examine item means, standard deviations, skewness (|skewness| < 2), and kurtosis (|kurtosis| < 7) to assess normality [38]. Item–total correlations were evaluated, with values < 0.30 considered for potential removal [39], and changes in Cronbach’s α if an item was deleted were also examined. The suitability of the data for EFA was assessed using the Kaiser–Meyer–Olkin (KMO) measure of sampling adequacy and Bartlett’s test of sphericity (*p* < 0.05) [31,39]. For the EFA, we used Principal Axis Factoring (PAF) as the factor extraction method to ensure appropriate psychometric validation of the latent construct, the item set and applied oblique rotation (Promax with Kaiser normalization; rotation parameter = 4) to allow correlated factors, consistent with the theoretical assumption that the four dimensions of moral resilience are interrelated [27,31]. Factor and item retention were determined using the following criteria: (a) eigenvalues, (b) communalities ≥ 0.40, (c) factor loadings ≥ 0.40, (d) item retention guided by communalities and salient pattern loadings, while monitoring cross-loadings, and (e) visual inspection of the scree plot [31,38,39]. These combined criteria ensured that both statistical and practical significance were considered in determining the factor structure.

Before conducting CFA (n = 209), univariate normality was assessed using skewness (|skewness| < 2) and kurtosis (|kurtosis| < 7) [38]. Multivariate normality was evaluated using Mardia’s normalized estimate, with the critical ratio (≥2.58, *p* < 0.01) used to determine violations of multivariate normality and the appropriate estimation method. Model fit was evaluated using multiple goodness-of-fit indices: chi-square minimum/degree of freedom (CMIN/df ≤ 2), standardized root mean square residual (SRMR ≤ 0.08), root mean square error of approximation (RMSEA ≤ 0.08), goodness-of-fit index (GFI ≥ 0.90), comparative fit index (CFI ≥ 0.90), Tucker–Lewis index (TLI ≥ 0.90), normed fit index (NFI ≥ 0.90), Hoelter’s Critical N, and modification indices for error terms [39]. Standardized factor loadings (≥0.50) and critical ratios (≥2.58, *p* < 0.01) were examined to determine whether items loaded significantly on their respective factors. Convergent validity in the CFA was assessed using average variance extracted (AVE ≥ 0.50) and construct reliability (≥0.70) [39].

Discriminant validity was evaluated by comparing the squared correlations between factors (Φ^2^) with the corresponding AVE values (Φ^2^ < AVE) and by examining whether the 95% confidence intervals of inter-factor correlations (Φ ± 2 × SE) included 1.00 [39]. In addition, given the relatively high correlations among some factors, the Heterotrait–Monotrait ratio (HTMT) was calculated, and 95% confidence intervals were estimated to confirm that all HTMT values were below the cutoff of 0.90 and that the confidence intervals did not include 1.00 [40], thereby providing a strengthened assessment of discriminant validity. Furthermore, alternative CFA models (reduced-factor models and a higher-order model) were compared to further evaluate the relative fit of the proposed four-factor structure.

Third, hypothesis testing for convergent validity was conducted using Pearson correlation analysis to examine the relationship between moral resilience, as measured by the K-RMRS-16™, and resilience, a theoretically related construct [9,41]. A correlation coefficient > 0.50 was considered indicative of adequate convergent validity [42]. Known-groups validity was tested based on the hypothesis that levels of moral resilience would differ according to levels of burnout [19,20]. Burnout scores were converted to T-scores and classified into three groups (low, moderate, and high) according to the criteria proposed by the instrument developer [33]. Differences in moral resilience across burnout groups were analyzed using one-way analysis of variance, followed by Scheffé post hoc tests or Welch’s test with Games–Howell post hoc comparisons, as appropriate (*p* < 0.05).

Finally, internal consistency reliability of the instrument was evaluated using Cronbach’s α, with values ≥ 0.70 considered acceptable [39].

## 3. Results

### 3.1. General Characteristics

The mean age of the 417 participants was 36.3 years, with nurses in their 30s accounting for the largest proportion (45.6%). Most participants were women (94.7%). More than half were married (54.0%), and the majority held a bachelor’s degree (72.7%). The mean total length of clinical experience was 12.07 years. The largest proportion of participants had 5–10 years of experience (33.8%), followed by 10–15 years (18.2%), 15–20 years (17.3%), <5 years (16.1%), and ≥20 years (14.6%).

The mean length of experience in the current department was 4.69 years. Specifically, 32.3% had 1–3 years of experience, 22.7% had ≥7 years, 17.1% had 3–5 years, 15.2% had <1 year, and 12.7% had 5–7 years. Most participants were staff nurses (80.1%), followed by specialty nurses (12.9%) and charge nurses (7.0%). Regarding work units, 60.7% worked in general wards, 26.6% in intensive care units or emergency departments, and 12.7% in operating or recovery rooms. With respect to hospital type, 64.5% worked in tertiary hospitals and 35.5% in general hospitals. Participants were distributed across regions as follows: Honam (30.7%), metropolitan area (28.8%), central region (21.6%), and the Gyeongsang region (18.9%).

There were no statistically significant differences between the EFA (n = 208) and CFA (n = 209) samples in terms of general characteristics (*p* > 0.05), indicating homogeneity between the two groups (Table 1).

### 3.2. Construct Validity

#### 3.2.1. Item Analysis

Item mean scores ranged from 2.50 to 3.34 (out of 4), with standard deviations ranging from 0.46 to 0.85. Absolute values of skewness ranged from 0.02 to 0.44, and absolute values of kurtosis ranged from 0.03 to 1.90, meeting commonly accepted criteria for normality [38]. Item–total correlation coefficients ranged from 0.43 to 0.64, with no items falling below the criterion of 0.30 [39]. Examination of Cronbach’s α values if items were deleted indicated that all 16 items were appropriate for inclusion in the EFA.

#### 3.2.2. EFA

EFA was conducted using data from 208 participants randomly assigned from the total sample. The Kaiser–Meyer–Olkin measure of sampling adequacy was 0.85, and Bartlett’s test of sphericity was significant (χ^2^ = 1467.61, *p* < 0.001), indicating that the data were suitable for factor analysis. Using PFA with an oblique rotation (Promax with Kaiser normalization; rotation parameter = 4), a four-factor solution was retained based on eigenvalues, scree plot inspection, and interpretability. Pattern loadings ranged from 0.51 to 0.86, and communalities (extraction h^2^) ranged from 0.40 to 0.70. The four factors accounted for 56.45% of the total variance based on extraction sums of squared loadings (Factor 1: 32.42%; Factor 2: 11.30%; Factor 3: 8.15%; Factor 4: 4.58%). Inter-factor correlations supported the use of an oblique rotation (r = 0.26~0.54), with the highest correlation observed between Relational integrity and Moral efficacy (r = 0.54) and between Personal integrity and Moral efficacy (r = 0.52) (Table 2).

#### 3.2.3. CFA

**Figure 1:**
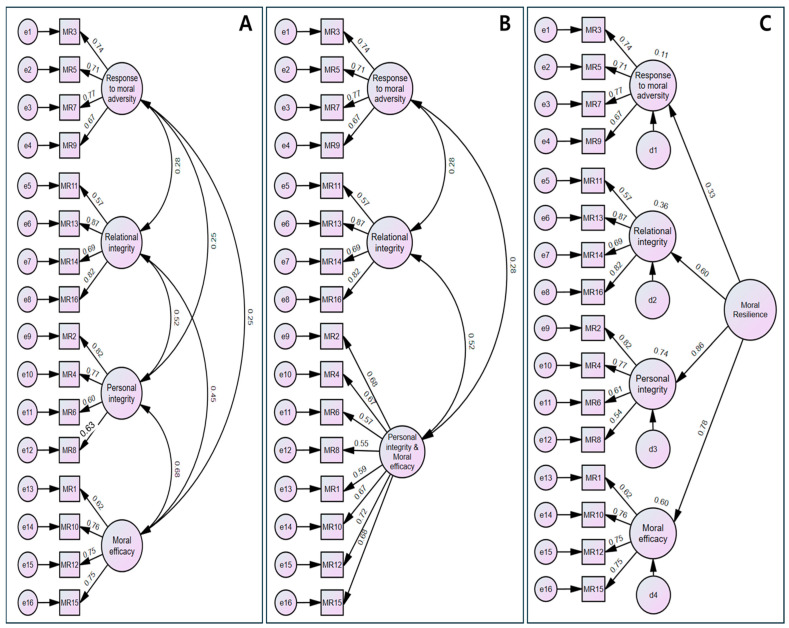
The proposed models of Korean version of the Rushton Moral Resilience Scale-16^TM^. (**A**) Model 1 (4 factor model). (**B**) Model 2 (3 factor model). (**C**) Model 3 (higher-order model).

CFA was conducted using data from 209 participants who were not included in the EFA sample. No missing data or outliers were identified. Univariate normality was supported, with absolute skewness values ranging from 0.01 to 0.34 and kurtosis values ranging from 0.12 to 2.05. Multivariate normality was not satisfied, as indicated by Mardia’s normalized estimate (critical ratio = 5.01); therefore, bootstrapped maximum likelihood estimation was used. Model fit indices indicated an adequate fit to the data: CMIN/df = 1.77, SRMR = 0.05, RMSEA = 0.06, GFI = 0.91, CFI = 0.94, TLI = 0.93, and NFI = 0.90. Hoelter’s Critical N at the 0.01 significance level was 160, and the sample size of 209 exceeded the recommended minimum (≥200) (Table 3). Standardized factor loadings ranged from 0.57 to 0.87, and all items loaded significantly on their intended factors (critical ratios ranged from 6.39 to 12.74; *p* < 0.001) (Table 3). To further evaluate the dimensionality of the scale, alternative CFA models were compared. The reduced-factor (3-factor) model showed inferior fit relative to the hypothesized model, whereas the higher-order model demonstrated comparable fit to the four-factor model (Table 3; Figure 1). Based on the overall pattern of fit indices, statistically significant factor loadings, and theoretical interpretability, the four-factor structure was retained as the final model without post hoc modification.

#### 3.2.4. Convergent and Discriminant Validity

Convergent validity was supported, with standardized factor loadings ranging from 0.57 to 0.87 (*p* < 0.001), AVE values ranging from 0.51 to 0.56, and construct reliability values ranging from 0.80 to 0.83, all meeting recommended criteria. Discriminant validity was examined using the Fornell–Larcker criterion and correlation confidence intervals. AVE values (0.51–0.56) exceeded squared inter-factor correlations (Φ^2^), which ranged from 0.06 to 0.46, and the 95% confidence intervals of inter-factor correlations (Φ ± 2 × SE) did not include 1.00 (Table 3).

Given the relatively high association observed for certain factor pairs, additional discriminant validity evidence was evaluated using the heterotrait–monotrait ratio (HTMT) with bootstrapped 95% confidence intervals (2000 resamples). HTMT values ranged from 0.308 to 0.621, and all bootstrapped confidence intervals remained below 1.00 (F1–F2: 0.48 [0.31~0.63]; F1–F3: 0.35 [0.18~0.51]; F1–F4: 0.31 [0.16~0.47]; F2–F3: 0.43 [0.29~0.56]; F2–F4: 0.62 [0.48~0.74]; F3–F4: 0.60 [0.45~0.72]), supporting acceptable discriminant validity of the four subscales (Table 3).

### 3.3. Hypothesis Testing for Convergent Validity

A positive correlation between moral resilience and resilience was hypothesized based on prior studies [9,41]. Pearson correlation analysis using data from all 417 participants demonstrated a significant positive correlation between moral resilience and resilience (r = 0.51, *p* < 0.001), exceeding the criterion for convergent validity (r > 0.50) [42] (Table 4).

### 3.4. Hypothesis Testing for Known-Groups Validity

Known-groups validity was examined based on the hypothesis that moral resilience would differ according to levels of burnout [19,20]. Burnout scores were converted to T-scores and categorized into low (≤43; 24.0%), moderate (44–56; 52.8%), and high (≥57; 23.2%) groups [33]. Moral resilience scores differed significantly across burnout groups (F = 71.92, *p* < 0.001), supporting known-groups validity (Table 4). Post hoc analyses indicated that moral resilience scores were highest in the low burnout group, followed by the moderate group, and lowest in the high burnout group.

### 3.5. Reliability

Internal consistency reliability of the final K-RMRS-16™ was satisfactory. Cronbach’s α was 0.86 for the total scale and ranged from 0.80 to 0.83 across the four subscales: response to moral adversity (0.83), relational integrity (0.80), personal integrity (0.83), and moral efficacy (0.81), all exceeding the recommended threshold of 0.70 (Table 4).

## 4. Discussion

We translated and culturally adapted the RMRS-16™ revised by Rushton et al. [27] for use in South Korean nursing practice and evaluated its validity and reliability among 417 nurses. The findings indicate that the K-RMRS-16™ is a psychometrically sound instrument for assessing nurses’ moral resilience in South Korean clinical settings.

Overall, the K-RMRS-16™ demonstrated robust validity and reliability across multiple forms of evidence, including internal consistency and content, structural, convergent, and known-groups validity. The original four-factor, 16-item structure was replicated in the EFA, and the cumulative variance explained was 56.45%, indicating acceptable explanatory power for a multidimensional psychometric scale [31]. CFA further supported model adequacy, and both convergent and discriminant validity met recommended criteria. Internal consistency was also satisfactory for the total scale (Cronbach’s α = 0.86) and for each subscale (α = 0.80~0.83). These results are comparable to those reported for the original RMRS-16™ (Cronbach’s α = 0.85~0.86) [27], as well as the Greek (α = 0.78) [28] and Japanese (α = 0.85) [29] versions, suggesting that the RMRS-16™ structure is applicable to the South Korean nursing setting. Collectively, these findings support moral resilience as a cross-culturally meaningful construct and underscore the applicability of a standardized measurement tool within South Korean nursing practice. Notably, correlations between some subscales were moderate to high, raising the possibility of partial conceptual overlap. To address this concern, we supplemented traditional discriminant validity evidence with HTMT estimates and bootstrapped confidence intervals. HTMT values (0.31~0.62) were below commonly used thresholds, and none of the bootstrapped confidence intervals included 1.00, supporting the distinctiveness of the four domains while acknowledging that certain facets of moral resilience may be closely related in clinical practice.

Hypothesis-testing construct validity was examined via convergent and known-groups validity [42]. Moral resilience showed a significant positive association with general resilience (r = 0.51), consistent with previous research [9,41]. This pattern suggests that moral resilience shares conceptual overlap with general resilience as a capacity to cope with adversity, while also reflecting a distinct construct that is specifically situated in moral and ethical contexts. Notably, the magnitude of correlation observed in the present study is similar to that reported in the original RMRS development study (r = 0.48) [9] and the Greek validation study (r = 0.53) [28], indicating that the relationship between moral resilience and general resilience may be relatively stable across cultures.

Known-groups validity was also supported: moral resilience scores differed significantly by burnout level, with the low-burnout group demonstrating higher moral resilience than the moderate- and high-burnout groups. This finding aligns with prior studies identifying moral resilience as a protective factor that may attenuate burnout-related outcomes [19,20]. These results provide empirical support for moral resilience as a potential target for interventions aimed at mitigating burnout associated with moral injury and distress. Accordingly, assessing moral resilience and implementing educational programs and organizational strategies to strengthen it may contribute to reducing burnout. Early identification of nurses experiencing high burnout and the provision of tailored psychosocial interventions may therefore be particularly beneficial.

During the translation and adaptation process, the study team proactively addressed potential cultural differences in the understanding of morality and ethical concepts that have been noted in prior cross-cultural validation work (e.g., the Japanese version) [29]. A systematic forward–backward translation approach was used, and iterative expert review was conducted to support measurement equivalence between the Korean and original versions. These procedures enhance the linguistic, conceptual, and cultural equivalence of the K-RMRS-16™. The validated K-RMRS-16™ may therefore facilitate the assessment of moral resilience levels among South Korean nurses and support evidence-based planning for educational and organizational interventions.

With regard to the subscales, response to moral adversity captures nurses’ emotional and behavioral reactions to ethical challenges [27]. Given the potential contribution of negative reactions such as helplessness, feeling overwhelmed, persistent distress, and excessive self-sacrifice to poorer mental health and increased burnout and turnover intention [9], strategies to mitigate these responses are warranted. Relational integrity reflects the ability to sustain moral beliefs while balancing interpersonal relationships in conflict situations [27]. In South Korean nursing organizations, hierarchical norms and relationship-oriented cultural expectations may coexist [35], which can make it difficult for nurses to express and maintain ethical positions in the face of authority and relational pressure. Nevertheless, fostering relational integrity is essential for nurses to effectively fulfill their professional role as patient advocates.

Personal integrity reflects consistent action aligned with one’s values and ethical standards despite adversity, representing a core element of moral resilience and the integration of moral and professional identity [9,27]. Moral efficacy reflects confidence in recognizing ethical issues, communicating effectively, and resolving ethical conflicts in ways consistent with one’s values [9,27], which may strengthen nurses’ ethical voice and promote active participation in clinical decision-making.

Importantly, the K-RMRS-16™ should not be used as a diagnostic tool to categorize nurses as having or not having moral resilience. Rather, it should be interpreted as an instrument to support self-understanding and guide individual growth and organizational capacity development [9,27]. Individual scores should not be used as absolute cutoffs for labeling or judgment; instead, they should be used to identify strengths and areas for enhancement and to inform tailored developmental strategies [27]. Meaningful and sustainable change in clinical practice requires not only individual-level efforts but also organizational commitment to fostering an ethical practice environment [15,43].

In this regard, strengthening moral resilience is most likely to be effective when educational initiatives for nurses are implemented alongside organizational strategies. At the individual level, nurses may develop moral resilience through self-reflection, case-based learning, emotion regulation, and mindfulness-based training [15,43]. At the relational level, interventions such as structured ethical dialogues, regular ethics rounds, reflective writing, and peer support groups may promote moral resilience. Building a culture of interprofessional collaboration and mutual respect may also reduce ethical isolation and strengthen collective moral capacity [15,43]. At the organizational level, consistent with ANA recommendations [15], healthcare organizations should invest in organizational infrastructures such as formalized training programs, active ethics committees, organizational support systems, and work environments that respect nurses’ ethical autonomy.

This study also suggests that moral resilience components are closely connected to nurses’ experiences of moral distress, conflict, and moral injury. Prior research, particularly from COVID-19 pandemic studies, has linked moral resilience to lower burnout and turnover, and improved healthcare service quality [16,19,20,43]. The present findings similarly suggest that strengthening moral resilience may contribute to nurses’ job satisfaction and psychological well-being, highlighting the importance of early recognition of moral distress and the provision of systematic psychosocial interventions. In this broader perspective, moral resilience may also be conceptualized in relation to other psychosocial constructs relevant to healthcare professionals, such as moral courage, ethical climate, professional identity, and psychological well-being, suggesting the value of integrative models in future research. In addition, moral resilience may be understood as part of a broader socio-emotional resource system that encompasses resilience, prosocial behavior, moral reasoning, and sense of coherence, which jointly support healthcare professionals’ capacity to cope with work-related stress and maintain psychological well-being.

Finally, this study indicates that the RMRS-16™ may serve as a standardized instrument for evaluating moral resilience and related ethical capacities among South Korean nurses and may provide foundational evidence for future clinical application, ethics education, and intervention development. However, several limitations should be noted. First, the sample consisted predominantly of female nurses, which reflects the gender distribution of the nursing workforce in South Korea but may limit the generalizability of the findings to male nurses. Second, owing to regional imbalance and convenience-based selection of institutions, nationwide representativeness was not fully ensured. Future studies should employ stratified or probability-based sampling strategies to enhance generalizability. Third, self-report measures may be susceptible to response bias, including social desirability. Fourth, given the cross-sectional design, a causal relationship between moral resilience and burnout cannot be established. In addition, future research should examine the factorial invariance of the K-RMRS-16™ across different subgroups (e.g., type of clinical unit or hierarchical level) and adopt longitudinal and intervention-based designs to further explore trajectories of moral resilience and its relationships with well-being and organizational variables across diverse clinical contexts in South Korea (e.g., emergency departments, intensive care units, and hospice settings).

## 5. Conclusions

In this study, we translated and culturally adapted the RMRS-16™ for use among South Korean nurses and evaluated its validity and reliability in a sample of 417 nurses. The findings demonstrate that the K-RMRS-16™ is a standardized and psychometrically sound instrument for assessing nurses’ moral resilience in South Korean clinical practice and provide empirical evidence that moral resilience is associated with lower levels of burnout.

The K-RMRS-16™ may serve as a foundational measurement tool to support future research, ethics education, and intervention development aimed at strengthening moral resilience among nurses. By enabling the systematic assessment of moral resilience, this instrument may contribute to advancing evidence-based strategies to promote nurses’ well-being and ethical practice within South Korean healthcare settings.

## Figures and Tables

**Table 1:** General characteristics of participants (n = 417).

Characteristics	Categories	Total (n = 417)	EFA (n = 208)	CFA (n = 209)	χ^2^ or t	*p*
Sex	Female	395 (94.7)	199 (95.7)	196 (93.8)	7.48	0.387
	Male	22 (5.3)	9 (4.3)	13 (6.2)		
Age (yrs)		36.3 ± 8.16	36.0 ± 8.02	36.5 ± 8.30	−0.54	0.591
	24–29	94 (22.5)	45 (21.6)	49 (23.4)	1.56	0.669
	30–39	190 (45.6)	101 (48.6)	89 (42.6)		
	40–49	100 (24.0)	47 (22.6)	53 (25.4)		
	≥50	33 (7.9)	26 (7.2)	18 (8.6)		
Marital status	Unmarried	192 (46.0)	96 (46.2)	96 (45.9)	0.00	0.964
	Married	225 (54.0)	112 (53.8)	113 (54.1)		
Level of	Diploma	35 (8.4)	15 (7.2)	20 (9.6)	0.99	0.610
education	Bachelor’s	303 (72.7)	155 (74.5)	148 (70.8)		
	≥Master’s degree	79 (18.9)	38 (18.3)	41 (19.6)		
Total clinical		12.07 ± 7.84	11.84 ± 7.71	12.30 ± 7.98	−0.60	0.274
career (yrs)	<5	67 (16.1)	29 (13.9)	38 (18.2)	4.47	0.346
	5–<10	141 (33.8)	80 (38.5)	61 (29.1)		
	10–15	76 (18.2)	37 (17.8)	39 (18.7)		
	15–<20	72 (17.3)	33 (15.9)	39 (18.7)		
	≥20	61 (14.6)	29 (13.9)	32 (15.3)		
Work experience		4.69 ± 4.70	4.62 ± 4.40	4.75 ± 4.99	−0.28	0.780
in present	<1	56 (15.2)	25 (12.0)	31 (14.8)	1.41	0.843
unit (yrs)	1–<3	142 (32.3)	71 (34.1)	71 (34.0)		
	3–<5	71 (17.1)	39 (18.8)	32 (15.3)		
	5–<7	54 (12.7)	26 (12.5)	28 (13.4)		
	≥7	94 (22.7)	47 (22.6)	47 (22.5)		
Position	Staff nurse	334 (80.1)	168 (80.8)	166 (79.4)	0.95	0.623
	Physician assistant	54 (12.9)	28 (13.4)	26 (12.5)		
	Charge nurse	29 (7.0)	12 (5.8)	17 (8.1)		
Working unit	Ward	253 (60.7)	124 (59.6)	129 (61.7)	1.10	0.577
	ICU & ER	111 (26.6)	54 (26.0)	57 (27.3)		
	OR & RR	53 (12.7)	30 (14.4)	23 (11.0)		
Hospital type	Advanced general	269 (64.5)	133 (63.9)	136 (65.1)	0.06	0.810
	General	148 (35.5)	75 (36.1)	73 (34.9)		
Region of	Capital	120 (28.8)	57 (27.4)	63 (30.1)	1.50	0.682
hospital	Central	90 (21.6)	43 (20.7)	47 (22.5)		
	Gyeongsang	79 (18.9)	44 (21.1)	35 (16.8)		
	Honam	128 (30.7)	64 (30.8)	64 (30.6)		

Values are presented as number (%) or mean ± standard deviation. CFA, confirmatory factor analysis; EFA, exploratory factor analysis; ER, emergency room; ICU, intensive care unit; OR, operation room; RR, recovery room.

**Table 2:** Exploratory factor analysis of the K-RMRS-16^TM^ (n = 208).

Factors/Items		ITC	Communality	Structure Matrix				Pattern Matrix			
		r		F1	F2	F3	F4	F1	F2	F3	F4
Response to moral adversity (F1)											
7	After facing a challenging ethical situation, lingering distress weighs me down. (R)	0.49	0.66	0.80	0.27	0.25	0.26	0.84	−0.12	0.12	−0.05
5	I am overwhelmed by persistent ethical conflicts. (R)	0.46	0.62	0.79	0.28	0.15	0.24	0.81	−0.02	−0.04	−0.02
9	When confronted with an ethical challenge, I push myself beyond what is healthy for me. (R)	0.53	0.61	0.77	0.38	0.20	0.34	0.72	0.09	−0.09	0.10
3	Difficult ethical situations leave me feeling powerless. (R)	0.49	0.52	0.72	0.35	0.22	0.23	0.70	0.07	0.03	−0.06
Relational integrity (F2)											
16	My fear can cause me to act in a way that compromises my values. (R)	0.58	0.69	0.32	0.82	0.40	0.26	−0.01	0.86	−0.05	−0.04
13	When others criticize my opinions, I compromise my values. (R)	0.64	0.66	0.36	0.80	0.45	0.36	0.02	0.77	−0.01	0.08
14	I would rather avoid conflict with those who have more authority than I do than act in accordance with my values. (R)	0.54	0.56	0.23	0.75	0.44	0.27	−0.09	0.75	0.06	0.00
11	I tend to be distracted by others’ strong emotions when ethical conflicts occur. (R)	0.60	0.50	0.16	0.39	0.82	0.43	0.28	0.51	0.00	0.01
Personal integrity (F3)											
4	I have the conviction to act in accordance with my values.	0.51	0.70	0.16	0.39	0.82	0.43	−0.04	−0.07	0.86	0.01
2	No matter the situation I do what is consistent with my values.	0.57	0.68	0.16	0.61	0.78	0.34	0.09	−0.12	0.69	0.12
8	I have the courage to take action when others resist.	0.50	0.51	0.26	0.33	0.70	0.46	−0.12	0.32	0.68	−0.10
6	I take responsibility for my choices.	0.54	0.41	0.30	0.44	0.62	0.38	0.12	0.09	0.53	0.02
Moral efficacy (F4)											
10	When I am confronted with an ethical challenge, I can articulate the ethical conflict.	0.43	0.57	0.24	0.27	0.33	0.76	−0.03	0.05	−0.11	0.81
12	I am confident in my ability to reason through ethical challenges in my professional role.	0.44	0.58	0.21	0.23	0.46	0.75	−0.05	−0.09	0.14	0.72
15	I can think clearly when confronting an ethical challenge, even when I feel pressured.	0.44	0.46	0.26	0.25	0.38	0.68	0.02	−0.02	0.03	0.66
1	I voice my ethical concerns in a way that others take seriously.	0.48	0.40	0.27	0.35	0.40	0.59	0.02	0.13	0.06	0.51
Eigenvalue				3.34	3.82	3.64	2.25				
% of variance				32.42	11.30	8.15	4.58				
Cumulative explained variance (%)				32.42	43.72	51.87	56.45				
Cronbach’s α				0.85	0.83	0.82	0.78				
Total Chronbach’s α = 0.87, Kaiser-Meyer-Olkin values = 0.85, Bartlett’s test of sphericity: χ^2^ = 1467.61 (*p* < 0.001)											
Inter-factor correlations: F1 ⟷ F2 (0.41), F1 ⟷ F3 (0.26), F1 ⟷ F4 (0.35), F2 ⟷ F3 (0.54), F2 ⟷ F4 (0.37), F3 ⟷ F4 (0.52)											

K-RMRS-16^TM^, Korean version of Rushton Moral Resilience Scale-16^TM^; ITC, item-total correlation; F, factor; R, reverse item.

**Table 3:** Findings of confirmatory factor analysis and item convergent-discriminant validity (n = 209).

**Convergent Validity**	**Items**		**Standardized** **Estimate (β)**		**Unstandardized** **Estimate (β)**	**SE**	**C.R.**	**AVE**	**CR**	**Cronbach’s α**
**Factors**										
Response to moral	9		0.67		1.00	-	-	0.52	0.81	0.81
adversity	7		0.77		1.10	0.13	8.80			
	5		0.71		0.98	0.12	8.37			
	3		0.74		0.98	0.11	8.62			
Relational integrity	16		0.82		1.00	-	-	0.56	0.83	0.82
	14		0.69		0.98	0.10	10.20			
	13		0.87		1.13	0.09	12.74			
	11		0.57		0.69	0.08	8.18			
Personal integrity	8		0.63		1.00	-	-	0.51	0.80	0.77
	6		0.60		0.96	0.15	6.39			
	4		0.77		1.30	0.18	7.31			
	2		0.82		1.32	0.18	7.48			
Moral efficacy	15		0.75		1.00	-	-	0.52	0.81	0.81
	12		0.75		0.97	0.10	9.89			
	10		0.76		0.97	0.10	9.98			
	1		0.62		0.67	0.09	8.19			
**Discriminant Validity**						**Φ (Φ^2^)**	**SE**	**Φ –** **(2 × SE)**	**Φ +** **(2 × SE)**	**HTMT [95% CI]**
**Relations**										
Response to moral adversity ⟷ Relational integrity						0.28 (0.08)	0.03	0.22	0.34	0.48 [0.31~0.63]
Response to moral adversity ⟷ Personal integrity						0.25 (0.06)	0.02	0.22	0.29	0.35 [0.18~0.51]
Response to moral adversity ⟷ Moral efficacy						0.25 (0.06)	0.02	0.21	0.29	0.31 [0.16~0.47]
Relational integrity ⟷ Personal integrity						0.52 (0.27)	0.02	0.48	0.56	0.43 [0.29~0.56]
Relational integrity ⟷ Moral efficacy						0.45 (0.20)	0.02	0.41	0.49	0.62 [0.48~0.74]
Personal integrity ⟷ Moral efficacy						0.68 (0.46)	0.02	0.64	0.72	0.60 [0.45~0.72]
**Fitness Index**		**CMIN/df**		**SRMR**		**RMSEA**	**GFI**	**CFI**	**TLI**	**NFI**
Model 1 (4 factors)		1.77		0.05		0.06	0.91	0.94	0.93	0.90
Model 2 (3 factors)		2.45		0.03		0.08	0.87	0.89	0.87	0.82
Model 3 (Higher-order)		1.76		0.03		0.06	0.91	0.94	0.93	0.90
Criteria		≤2		≤0.08		≤0.08	≥0.90	≥0.90	≥0.90	≥0.90

K-RMRS-16^TM^, Korean version of Rushton Moral Resilience Scale-16^TM^; AVE, average variance extracted; CMIN/df, chi-square minimum/degree of freedom; CR, construct reliability; C.R., critical ratio; SE, standard error; Φ, correlation coefficient; Φ^2^, squared correlation coefficient; HTMT, heterotrait-monotrait ratio; 95% CI, bootstrapped 95% confidence intervals (2000 resamples); CFI, comparative fit index; GFI, goodness of fit index; NFI, normed fit index; RMSEA, root mean square error of approximation; SRMR, standardized root mean square residual; TLI, Turker–Lewis index; Total Cronbach’s α = 0.85.

**Table 4:** Hypothesis-testing convergent validity and known-groups validity for the K-RMRS-16^TM^ (n = 417).

Variable	Sub-Factors or Categories		Moral Resilience				
			Response to Moral Adversity	Relational Integrity	Personal Integrity	Moral Efficacy	Total
Moral Resilience	Response to moral adversity		1				
	Relational integrity		0.32 (<0.001)	1			
	Personal integrity		0.25 (<0.001)	0.47 (<0.001)	1		
	Moral efficacy		0.25 (<0.001)	0.36 (<0.001)	0.52 (<0.001)	1	
	Total		0.50 (<0.001)	0.64 (<0.001)	0.58 (<0.001)	0.51 (<0.001)	1
Resilience (Mean ± SD: 3.89 ± 0.46)			0.32 (<0.001)	0.31 (<0.001)	0.35 (<0.001)	0.40 (<0.001)	0.51 (<0.001)
Mean ± SD			2.78 ± 0.57	2.81 ± 0.65	3.05 ± 0.46	2.91 ± 0.40	2.89 ± 0.38
Cronbach’s α			0.83	0.83	0.80	0.81	0.86
Burnout (T-score)	Low (≤43) ^a^	100 (24.0%)	3.02 ± 0.59	3.12 ± 0.64	3.24 ± 0.53	3.09 ± 0.41	3.16 ± 0.43
	Moderate (44–56) ^b^	220 (52.8%)	2.82 ± 0.49	2.83 ± 0.60	3.09 ± 0.37	2.95 ± 0.32	2.90 ± 0.26
	High (≥57) ^c^	97 (23.2%)	2.41 ± 0.56	2.45 ± 0.60	2.78 ± 0.45	2.63 ± 0.43	2.57 ± 0.30
	F (p)		29.72 (<0.001) †	29.99 (<0.001) *	24.89 (<0.001) †	31.93 (<0.001) †	71.92 (<0.001) †
	Post hoc		a > b > c	a > b > c	a > b > c	a > b > c	a > b > c

K-RMRS-16^TM^, Korean version of the Rushton Moral Resilience Scale-16^TM^; Values are presented as r (p), number (%), or mean ± standard deviation, unless otherwise stated. * F, one-way analysis of variance and post hoc Scheffé; † F, Welch analysis of variance and post hoc Games-Howell test.

## Data Availability

The data presented in this study are available on request from the corresponding author due to ethical and privacy considerations related to the protection of participants’ confidentiality.
